# Hierarchical power control of a large-scale wind farm by using a data-driven optimization method

**DOI:** 10.1371/journal.pone.0291383

**Published:** 2023-09-14

**Authors:** Pengyu Di, Xiaoqing Xiao, Feng Pan, Yuyao Yang, Xiaoshun Zhang

**Affiliations:** 1 Guangdong Power Grid Co., Ltd, Guangzhou, China; 2 Metrology Center of Guangdong Power Grid Co., Ltd, Qingyuan, China; 3 Foshan Graduate School of Innovation, Northeastern University, Foshan, China; National Institute of Technology Silchar, India, INDIA

## Abstract

With the participation in automatic generation control (AGC), a large-scale wind farm should distribute the real-time AGC signal to numerous wind turbines (WTs). This easily leads to an expensive computation for a high-quality dispatch scheme, especially considering the wake effect among WTs. To address this problem, a hierarchical power control (HPC) is constructed based on the geographical layout and electrical connection of all the WTs. Firstly, the real-time AGC signal of the whole wind farm is distributed to multiple decoupled groups in proportion of their regulation capacities. Secondly, the AGC signal of each group is distributed to multiple WTs via the data-driven surrogate-assisted optimization, which can dramatically reduce the computation time with a small number of time-consuming objective evaluations. Besides, a high-quality dispatch scheme can be acquired by the efficient local search based on the dynamic surrogate. The effectiveness of the proposed technique is thoroughly verified with different AGC signals under different wind speeds and directions.

## 1. Introduction

In respond to the energy shortage and environment degradation, the renewable energy like solar energy [1] has gained a rapid development in recent years around the world. As the backbone of renewable energy, the global new installed capacity of wind energy has grown remarkably, where the year-over-year growth is more than 50% from 2019 to 2020; and the compound annual growth rate of the offshore wind energy will be more than 30% from 2021 to 2025 [2]. In general, the power output of renewable energy is highly influenced by the weather conditions, such as wind speed, solar irradiance and temperature [3]. With the rapid increasing renewable energy, the power system is facing tremendous pressure on the real-time power balance due to the decreased moment of inertia and the increased random power fluctuation [4]. Hence, it requires more and faster reserve resources to participate in automatic generation control (AGC).

Since a large-scale wind farm (WF) can provide a sufficient regulation capacity and a superior regulation performance, extensive studies on AGC have been investigated by considering the deep participation with WF. To improve the dynamic regulation performance of the control area, a lifelong learning [5] was proposed for coordinated control between WFs and other AGC units. In [6], an adaptive distributed auction-based algorithm was presented to achieve a distributed optimization of AGC dispatch. Based on the similar control framework, a deep reinforcement learning [7] was designed to coordinate WFs and other energy resources in an integrated energy system. All of these works mainly focused on the control process of the whole system, in which a WF was regarded as a single wind turbine (WT). In fact, a WF consists of numerous WTs, where the AGC signal of the whole WF should be distributed to each WT.

From the viewpoint of WF, a set-point tracking control [8] was designed for AGC, in which the power command of each WT is proportional to its available power capacity. This simple proportional dispatch method can not satisfy the optimal control requirement of WF for AGC. To handle this issue, a distributed low-complexity controller based set-points optimization [9] was presented for fatigue reduction of WF. In [10], a distributed model predictive control was proposed for minimization of the WT load and improving the tracking performance of WF. Besides, an alternating direction method of multipliers (ADMM) based hierarchical optimal power control [11] was presented to guarantee the rotor speed stability, minimize the energy loss, and damp the oscillations. In [12], the distributed reactive power control can be achieved for the large-scale WF cluster by ADMM. Like the proportional dispatch, a leader-follower consensus protocol based on the fair load-sharing [13] was employed to realize a distributed control of power output regulation for WF. Similarly, a multi-agent framework based cooperative output regulation [14] was designed for a large-scale WF following leader-follower consensus control. Moreover, an efficient distributed economic model predictive control strategy [15] was proposed to take the power tracking and economic operation into account for a large-scale WF. To effectively reduce the optimization complexity and difficulty, the clustering algorithm [16] is usually used for dividing the large amount of WTs. In [17], an incremental clustering based equivalent method was proposed to improve the equivalent accuracy for the large-scale WFs. In [18], the large-scale WFs were classified into multiple groups by using a dynamic strategy, thus the real-time dispatch can be easily solved based on the grouping results. Based on the WTs clustering, a hierarchical power dispatch [19] was designed for a large-scale WF based on the proportional control and proportional distribution strategy. In [20], the hierarchical power control was extended to multiple large-scale WFs with active and reactive power control concurrently. However, these works did not consider the interactive influence between different WTs by the wake effect [21], i.e., the inlet wind speed of the downstream WTs can be influenced by the operating state of the upstream WTs.

By taking the wake effect into account, various methods [22] were proposed for power control of WF, including particle swarm optimization (PSO) [23], Bayesian Ascent based data-driven optimization [24], model-predictive optimization [25], cooperation-driven distributed control [26,27], double-layer machine learning [28], and so on. Most of these studies essentially belong to a single-degree-of-freedom control based dispatch, where the optimization space is restricted since the rotor speed control (RSC) and pitch angle control (PAC) are separated. To simultaneously implement these two control degrees, a small-signal method based two-degree-of-freedom control was constructed in [29], which can reduce the fatigue load during the power tracking. Following the same manner, a clustering-based coordinated control method [30] was proposed for power system frequency support by combining the kinetic energy, but it did not address the power tracking via distributing the AGC signal.

On the whole, the above studies did not consider the dynamic regulation performance in the control objective, i.e., a WF should trace the AGC signal as quickly and accurately as possible. In general, the response speed of RSC is faster than that of PAC [31], but the regulation range of WT power output by RSC is much narrower than that by PAC. Consequently, the control commands of these two degrees for all the WTs should be optimized to acquire a satisfactory dynamic regulation performance under different AGC signals. Meanwhile, it easily leads to a high optimization difficulty and an expensive computation due to the large number of controllable variables. To resolve these problems, this paper constructs a new hierarchical power control (HPC) for AGC signal tracking of WF. Like the conventional power dispatch optimization in a WF [23], the control command optimization of each group in HPC is a non-linear and non-convex problem. Compared with the gradient-based algorithms (e.g., the interior point method), the gradient-free algorithms are more competitive and flexible for this optimization as they are highly independent on the specific model of HPC with wake effect. As the most common gradient-free algorithms, genetic algorithm (GA) [32] and PSO [33] have been successfully applied to the optimal power dispatch in WF due to their high global searching ability and easy implementation. Naturally, it can be solved by other meta-heuristic based gradient-free algorithms. But the meta-heuristic algorithms easily result in a long computation time for a high-quality optimum because they should require adequate fitness evaluations. Hence, they are hard to achieve the real-time optimization of HPC with control time cycle from several seconds to minutes, especially for the large-scale WF with numerous WTs by considering the wake effect.

In recent years, the surrogate-assisted optimization [34] has become a popular way to avoid a high computation cost resulted from the large amount of evaluations on the objective or constraint functions. It usually approximates the objective or constraints functions by machine learning models based on the small amount of evaluation data [35], thus a fast optimization can be implemented by different algorithms based on the surrogate model. Inspired by this advantage, a data-driven surrogate-assisted optimization (DDSO) is employed for HPC. Compared with the existing studies, this work has two novelties, as follows:

- For the power control of a large-scale WF, most of the existing studies were carried out based on a single-degree-of-freedom control based dispatch, where the optimization space is restricted since RSC and PAC are separated. In [29,30], the constructed two-degree-of-freedom control can simultaneously implement these two control degrees, but they did not consider the regulation performance difference between RSC and PAC. As a result, it easily result in an unsatisfactory regulation performance for AGC. In contrast, the regulation performance difference between RSC and PAC is fully consider in this work, which can dramatically improve the dynamic regulation performance of the whole WF for AGC via two-degree-of-freedom control of rotor speed and pitch angle.
- Compared with the direct power control framework [28], the constructed HPC can effectively the optimization complexity and dimension due to the negligible wake interaction between different WT groups. Moreover, the presented DDSO can result in a much low computation cost against to the meta-heuristic algorithms [23] since it can reduces the number of fitness function evaluations. As a result, a high-quality AGC dispatch scheme can be rapidly acquired for a large-scale WF.

The rest of this work is organized as follows: Section 2 introduces the control framework and optimization model of HPC. The optimization principle and design by DDSO are provided in Section 3. Section 4 gives the simulation results and analysis, and Section 5 concludes this work.

## 2. Framework and optimization model of HPC

### 2.1. Control framework

**Fig 1:**
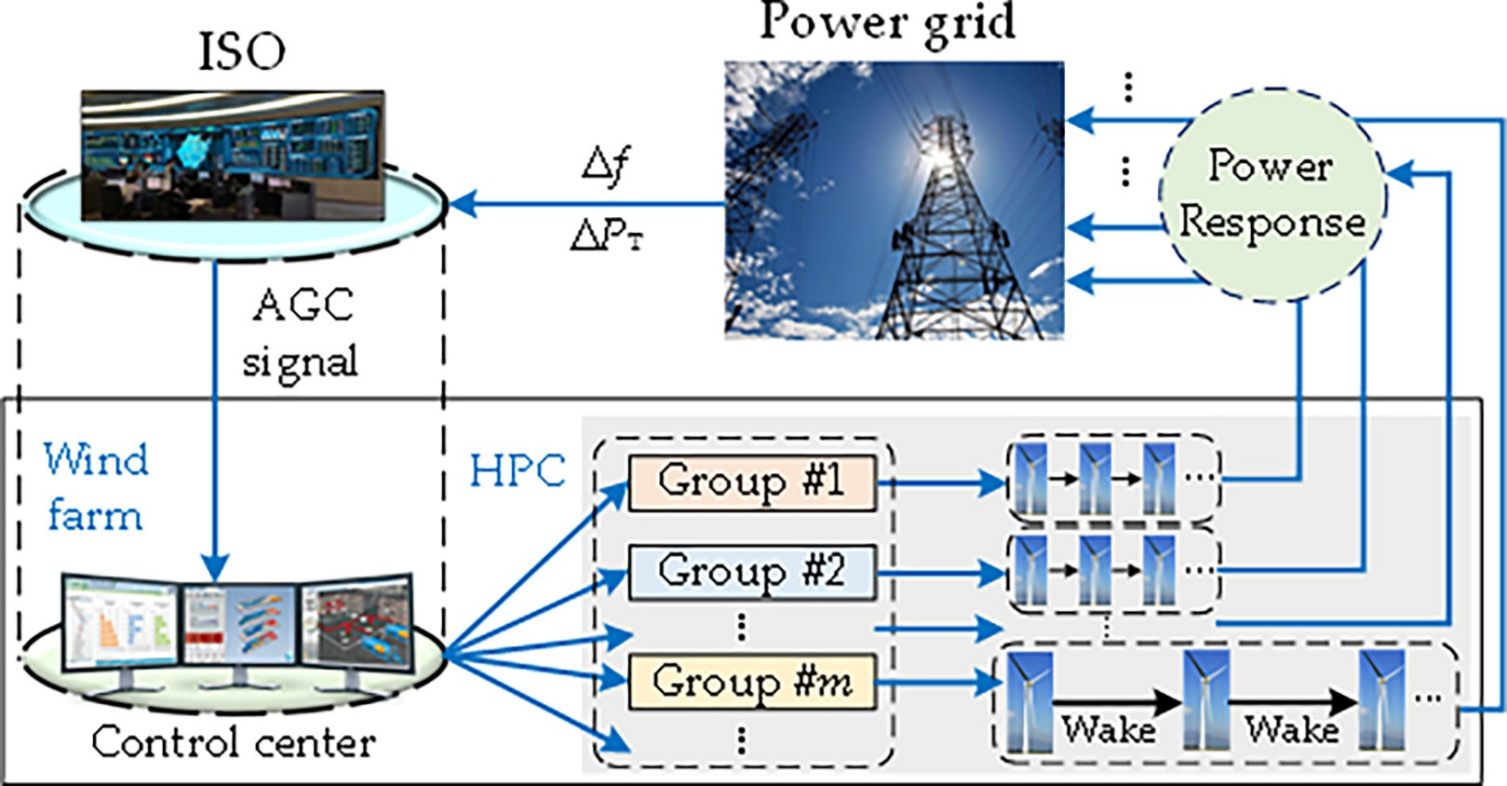
Control framework of hierarchical power control in a large-scale WF.

To achieve a dynamic power balance between supply and demand, the independent system operator (ISO) should implement the AGC for the control area based on the real-time deviations of frequency Δ*f* and tie-line exchange power Δ*P*_T_ with other control areas [36], as shown in Fig 1. In each control interval, the controller of AGC will generate the total dispatch command, then ISO will distribute it to different reserve resources. In respond to the AGC signal from ISO, the control center of a WF will carry out two operations. Firstly, the AGC signal of the whole WF will be distributed to different groups in proportion of their regulation capacities. Then the AGC signal of each group will be balanced by the corresponding WTs via searching the optimal rotor speeds and pitch angles.

### 2.2. WT model

Based on the aerodynamic rule, the energy conversion of the *i*th WT under different wind speeds can be described as

$$P_{i}=0.5\rho \pi R^{2}C_{p,i}(\lambda_{i},\beta_{i})V_{i}^{3}$$ (1)

$$F_{T,i}=0.5\rho \pi R^{2}C_{T,i}(\lambda_{i},\beta_{i})V_{i}^{2}$$ (2)

$$\lambda_{i}=\frac{\omega_{r,i}R}{V_{i}}$$ (3)

where *P*_*i*_ and *F*_T,*i*_ are the mechanical power and the thrust force of the *i*th WT, respectively; *ρ* is the air density; *R* denotes the rotor radius; *λ*_*i*_ and *β*_*i*_ are the tip speed ratio and the pitch angle of the *i*th WT, respectively; *V*_*i*_ is the inlet wind speed of the *i*th WT; *ω*_r,*i*_ is the rotor speed of the *i*th WT; *C*_p,*i*_ and *C*_T,*i*_ are the power coefficient and the thrust coefficient of the *i*th WT, respectively, which can be acquired via the look-up tables or the curves fitting [37].

**Fig 2:**
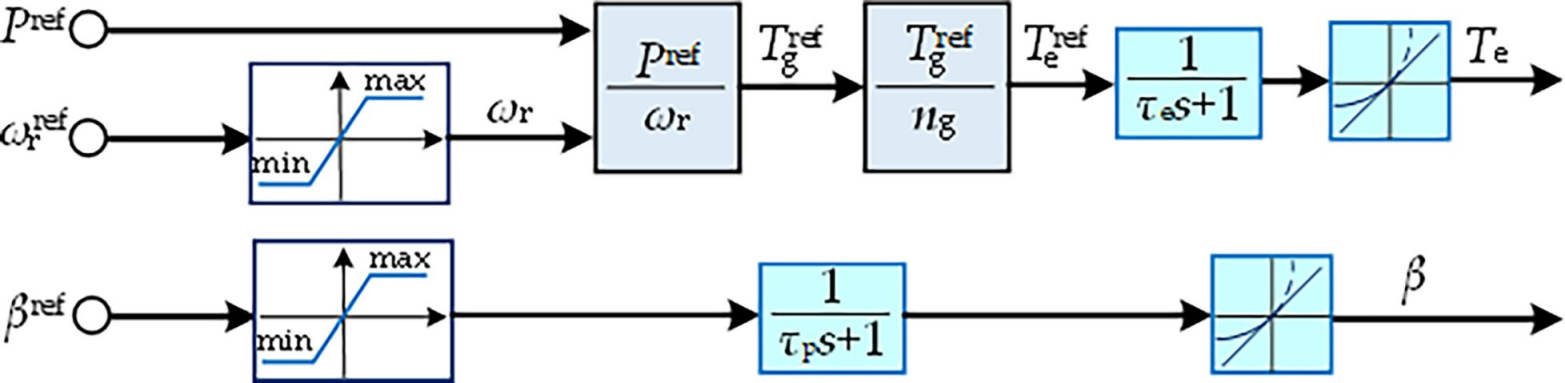
Schematic diagram of two-degree-of-freedom control for each WT.

Besides, the two-degree-of-freedom control framework [29] for each WT is given in Fig 2. The control inputs are assigned from the control center of WF, which includes reference active power *P*^ref^, reference rotor speed $\omega_{r}^{ref}$, and reference pitch angle *β*^ref^. The control outputs contain the electromagnetic torque of generator *T*_e_ and the pitch angle *β*. The control process is described by a first-order inertia transfer function with a bound limiter and a ramp constraint, where $T_{g}^{ref}$ and $T_{e}^{ref}$ are the reference generator torque and electromagnetic torque, respectively; *n*_g_ is the gear box ratio; *τ*_e_ and *τ*_p_ are the time constants for the generator and the pitch angle servo system, respectively.

### 2.3. Wake effect

**Fig 3:**
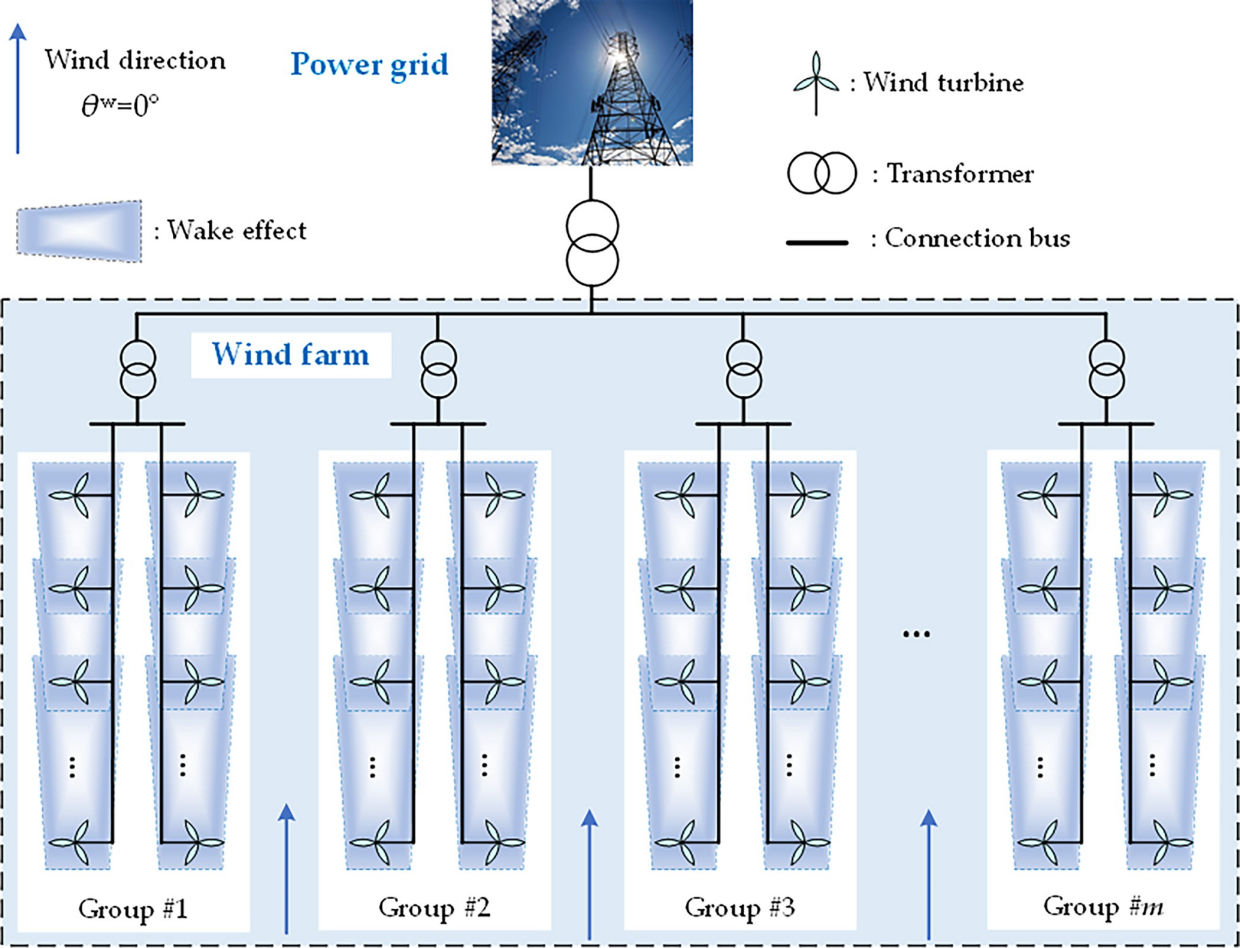
Illustration of group division of WTs in a large-scale wind farm.

When the wind inflow goes through the rotated blades, the wind speed behind the rotor will be reduced. Because of this wake effect, the wind speed input of the downstream WTs will be influenced by the operating states of the upstream WTs, as shown in Fig 3. To describe this influence, the basic Jensen model is adopted to evaluate the downstream wind speed, as follows [38]:

$$V_{x}=V_{in}\left[1-\frac{1-\sqrt{1-C_{T}}}{{\left(1+2kx/D\right)}^{2}}\right]$$ (4)

where *V*_in_ is the inlet wind speed of the upstream WT; *V*_*x*_ is the downstream wind speed with the distance *x* directly behind the upstream WT; *C*_T_ is the thrust coefficient of the upstream WT; *D* is the rotor diameter; and *k* is the wake decay constant.

In general, a downstream WT is easily influenced by multiple upstream WTs. By considering the shadowed area by the upstream WTs, the inlet wind speed of the *i*th WT can be calculated based on the Jensen’s multiple wake model, as [39]

$$V_{i}=\sqrt{V_{0}^{2}+\sum_{j=1}^{i-1}\alpha_{ij}(V_{ij}^{2}-V_{j}^{2})}$$ (5)

$$V_{ij}=V_{j}\left[1-\frac{1-\sqrt{1-C_{T,j}}}{{\left(1+2kD_{ij}/D\right)}^{2}}\right]$$ (6)

$$\alpha_{ij}=\frac{4S_{ij}}{\pi D^{2}}$$ (7)

where *V*_0_ is the natural wind speed; *V*_*ij*_ denotes the wake wind speed of the *j*th upstream WT at the *i*th WT location; *α*_*ij*_ denotes the intersection weight of the shadowed area at the *i*th WT location by the *j*th upstream WT; *D*_*ij*_ denotes the distance along the wind direction between the *i*th and *j*th WTs; and *S*_*ij*_ is the the shadowed area at the *i*th WT location by the *j*th upstream WT.

Note that *D*_*ij*_ and *S*_*ij*_ are associated with the dynamic wind direction and the fixed locations of WTs, which can be updated through the coordinate rotation [40] and the overlap evaluation [41] under different wind directions.

### 2.4. Group division of WTs

In this work, the WTs with wake interaction are clustered into a group, while different groups can be decoupled without wake interactions. Based on the wake effect described in Eqs (5)–(7), a large intersection weight *α*_*ij*_ indicates a strong wake effect from the *j*th upstream WT to the *i*th WT. Hence, two WTs can be put in the same group when their intersection weight is large than a certain value. To simplify this process, the WTs connected in the same transformer are divided into the same group [42] due to their short geographical distances and large shadowed areas, as illustrated in Fig 3.

### 2.5. Optimization model

#### 2.5.1 Objective function

In response to the AGC signal, the primary task of HPC is to trace the AGC signal as accurately and quickly as possible. Firstly, the power output of all the WTs should be equal to the AGC signal. Secondly, the variations of pitch angles should be as small as possible due to the slow response speed of RSC. Therefore, the objective function of HPC consists of two components, including the power deviation and pitch angle variation, as follows:

$$min\;f=\mu_{1}\Delta P_{WF}^{D}+\mu_{2}\Delta \beta_{WF}^{D}$$ (8)

$$\left\{ \begin{array}{c} \Delta P_{WF}^{D}=|P_{WF}^{ref}(k)-\sum_{i=1}^{n}P_{i}(k)|\\ \Delta \beta_{WF}^{D}=\sum_{i=1}^{n}{\left[\beta_{i}(k)-\beta_{i}(k-1)\right]}^{2} \end{array}\right.$$ (9)

where *f* is the overall objective function; *μ*_1_ and *μ*_2_ are weight coefficients for the power deviation and the pitch angle variation, respectively; $\Delta P_{WF}^{D}$ denotes the power variation of the whole WF; $\Delta \beta_{WF}^{D}$ denotes the pitch angle variation of the whole WF; $P_{WF}^{ref}$ is the AGC signal of the whole WF; *k* denotes the *k*th control interval of AGC; and *n* is the number of WTs in a WF.

#### 2.5.2 Constraints

Except the constraints of wake effect in Eqs (4)–(7), the constraints of HPC mainly include the power capacity constraint, operating constraints of pitch angle and rotor speed, as follows [43,44]:

$$0\leq P_{i}\leq P_{rate},i=1,2,\cdots ,n$$ (10)

$$\beta_{min}\leq \beta_{i}\leq \beta_{max},i=1,2,\cdots ,n$$ (11)

$${\left(\frac{d\beta }{dt}\right)}_{min}\leq \frac{d\beta_{i}}{dt}\leq {\left(\frac{d\beta }{dt}\right)}_{max},i=1,2,\cdots ,n$$ (12)

$$\omega_{min}\leq \omega_{r,i}\leq \omega_{max},i=1,2,\cdots ,n$$ (13)

where *P*_rate_ is the rated power of each WT; *β*_min_ and *β*_max_ are the minimum and maximum pitch angles, respectively; ${\left(\frac{d\beta }{dt}\right)}_{min}$ and ${\left(\frac{d\beta }{dt}\right)}_{max}$ are the minimum and maximum ramp rates of the pitch angle, respectively; *ω*_min_ and *ω*_max_ are the minimum and maximum rotor speeds, respectively.

## 3. Optimization principle and design of DDSO

### 3.1 Optimization principle of DDSO

**Fig 4:**
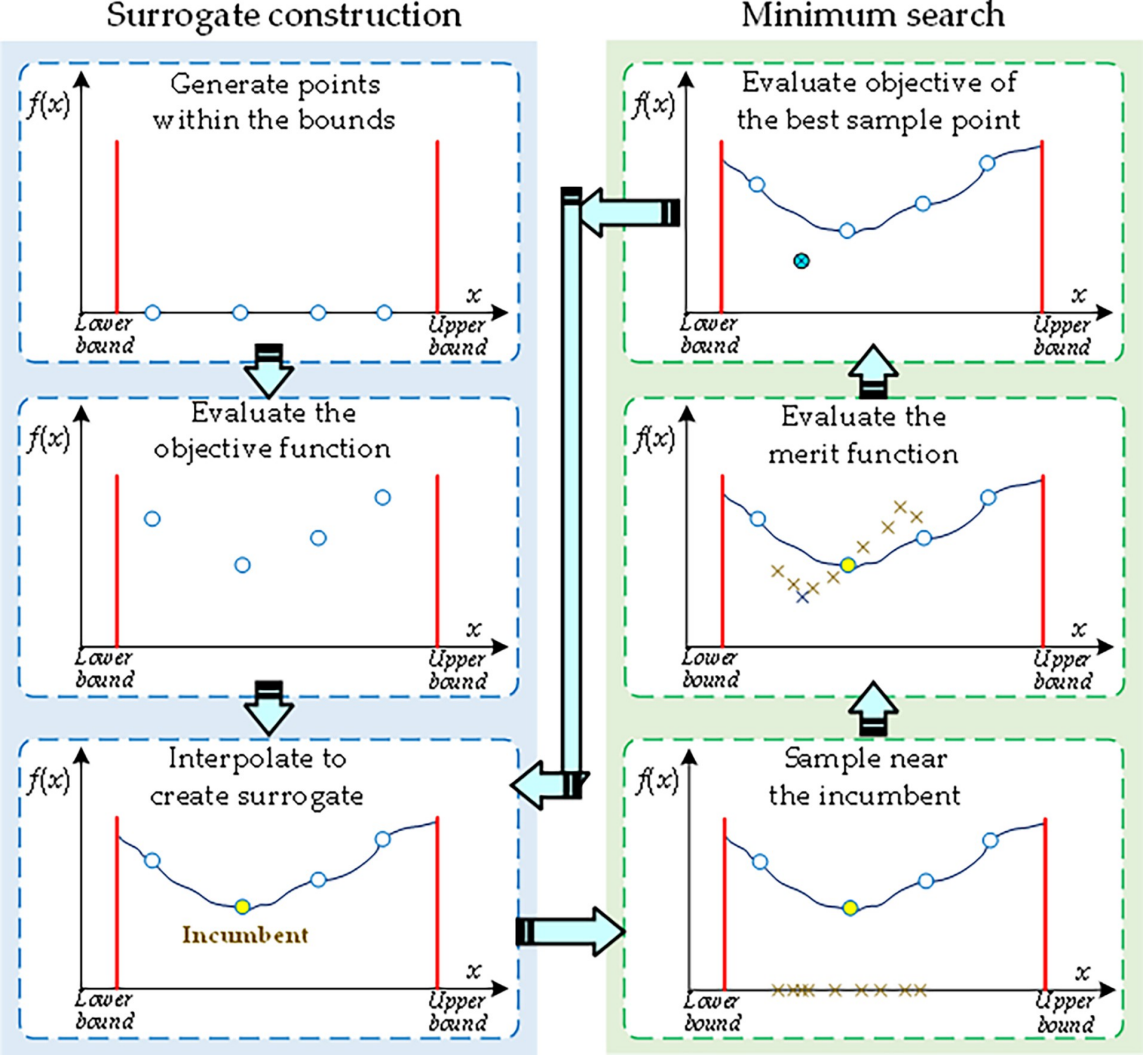
Operation process of DDSO.

In this work, the presented DDSO consists of two main operations, including the surrogate construction and minimum search [45], as shown in Fig 4. Firstly, the surrogate can be constructed by a radial basis function (RBF) interpolator based on the sample points. Secondly, multiple new solutions can be directly generated and evaluated through the local search based on current surrogate, in which the objective value of the best sample point will be evaluated and used for updated the surrogate. Therefore, a high-quality optimum can be acquired after multiple iterations with these two operations, while a high computation cost can be effectively avoided since the surrogate can accurately replace the original objective function.

#### 3.1.1 Surrogate construction

At the initial stage, the sample points are generated by a quasirandom sequence because it can quickly cover the optimization space more evenly than the pure random points. To satisfy the operating constraints in Eqs (10)–(13), the random sample points can be scaled and shifted to limited within the lower and upper bounds. Here, a frequently-used quasirandom sequence called Halton sequence is adopted to generate the initial sample points, as follows [46]:

$$x_{p}=(\Phi_{b_{1}}(p),\Phi_{b_{2}}(p),\cdots ,\Phi_{b_{d}}(p)),p=1,2,\cdots ,n_{0}$$ (14)

where ***x***_*p*_ is the *p*th sample point; $\Phi_{b_{d}}(p)$ represents a van der Corput sequence with a prime power base *b*_*d*_ for the *d*th dimension; *d* is the number of dimensions; and *n*_0_ is the number of initial random sample points.

Based on the generation solutions in Eq (14), the objective function of each solution can be evaluated according to Eqs (8) and (9) with the wake effect. Given these data points {(***x***_*p*_, *f*_*p*_)|*p* = 1,2,⋯,*n*_0_}, a RBF interpolator is employed to construct the surrogate due to its high flexibility and computation efficiency. Hence, the surrogate can be described as follows [47]:

$$s(x)=\sum_{p=1}^{n_{0}}\lambda_{p}\phi ({\left\|x-x_{p}\right\|}_{2})+\rho (x)$$ (15)

where *λ*_*p*_ denote the RBF coefficient for the *p*th point; ‖∙‖_2_ is the Euclidean norm; *ϕ* represents the radial function; and *ρ*(***x***) denotes the polynomial function for ***x***.

Here, a cubic radial function and a linear polynomial function are selected to construct the surrogate in Eq (15), as follows [48]:

$$\phi (r)=r^{3}$$ (16)

$$\rho (x)=c^{T}x+c_{0}$$ (17)

where ***c***∈ℝ^*d*^ denotes the coefficient vector of the linear term and *c*_0_ is the constant term.

With the sample data points, the unknow coefficients in Eq (15) can be determined through solving the linear equations, as follows [47]:

$$\left[\begin{array}{c} \lambda \\ c_{p} \end{array}\right]={\left[\begin{array}{cc} \psi & X^{T}\\ X & 0 \end{array}\right]}^{-1}\left[\begin{array}{c} F\\ 0 \end{array}\right]$$ (18)

$$X=\left[\begin{array}{ccc} x_{1}^{T} & \cdots & x_{n_{0}}^{T}\\ 1 & \cdots & 1 \end{array}\right]$$ (19)

where $\lambda ={\left[\lambda_{1},\lambda_{2},\cdots ,\lambda_{n_{0}}\right]}^{T}$ is the RBF coefficient vector; ***c***_p_ = [***c***^T^ *c*_0_]^T^ is the coefficient vector of the linear polynomial function; *ψ* is a *n*×*n* matrix with $\psi_{ij}=\phi ({\left\|x_{i}-x_{j}\right\|}_{2})$; and $F={\left[f_{1},f_{2},\cdots ,f_{n_{0}}\right]}^{T}$ is the objective function vector.

Note that the surrogate can be updated with the added sample points, while the unknow coefficients in Eq (18) will be unique if the inverse matrix is nonsingular [47].

#### 3.1.2 Minimum search

To achieve an efficient optimization, a local search based on the best point (i.e., incumbent point) found so far since the last reset of surrogate is implemented, as follows [48]:

$$x_{new}^{q}=\left\{ \begin{array}{l} x_{best}^{q},\;if\;\varepsilon >\varepsilon_{0}\\ x_{best}^{q}+r_{nd}\Delta \delta (x_{up}^{q}-x_{low}^{q}),else \end{array}\right.$$ (20)

$$\varepsilon_{0}=\left\{ \begin{array}{c} \max\left\{0.1,5/d\right\},\mathrm{if}\;d>5\\ 1,\;\mathrm{else} \end{array}\right.$$ (21)

$$r_{nd}∽N(0,1)$$ (22)

where $x_{new}^{q}$ denotes the value of the *q*th dimension for the new candidate solution,$q=1,2,\cdots ,d;\;x_{best}^{q}$ denotes the value of the *q*th dimension for the incumbent point; *r*_nd_ is a random value following the normal distribution N(0,1); *ε* is a random value uniformly distributed from 0 to 1; *ε*_0_ denotes the perturbation probability; $x_{up}^{q}$ and $x_{low}^{q}$ are the upper and the lower bounds of the *q*th dimension, respectively; and Δ*δ* is the search scale, which will be doubled after three successful searches and be halved after *d* unsuccessful searches since the last update.

Note that each candidate solution in Eq (22) should be limited within its lower and upper bounds after a perturbation based on the incumbent point. In this work, the number of candidate solutions is set to be 500*d* at each iteration. To avoid trapping into a low-quality optimum, a merit function is used to evaluate the candidate solutions instead of only using the surrogate. Particularly, it is a linear weighted sum of the scaled surrogate and the scaled distance, as follows [49]:

$$f_{merit}(x)=\mu S(x)+(1-\mu )L(x)$$ (23)

$$S(x)=\frac{s(x)-s_{min}}{s_{max}-s_{min}}$$ (24)

$$L(x)=\frac{l_{max}-l(x)}{l_{max}-l_{min}}$$ (25)

where *μ* is the weight coefficients of scaled surrogate, 0≤*μ*≤1; *S*(***x***) denotes the scaled surrogate; *L*(***x***) denotes the scaled distance; *s*_max_ and *s*_min_ are the maximum and minimum of surrogate among all the candidate solutions, respectively; *l*_max_ and *l*_min_ are the maximum and minimum distances among all the candidate solutions, respectively; and *l*(***x***) denotes the distance function between different candidate solutions.

As a result, a candidate solution with the smallest merit function will be chosen as the sample point at the next iteration, i.e., $x_{can}^{best}=\underset{p=1,2,\cdots ,500d}{\mathrm{argmin}}f_{merit}(x_{can}^{p})$. It will be evaluated by the original objective function $f(x_{can}^{best})$, and then used to update the surrogate.

As a whole, the execution procedure of DDSO is illustrated in Algorithm 1, where *k*_max_ is the maximum iteration number; $l_{max}^{in}$ is the maximum distance between all the current candidate solutions and the incumbent point; and *l*_reset_ is the tolerance distance to stop the minimum search and reset the surrogate.

**Algorithm 1:** Execution procedure of DDSO

**Input:** The objective function, constraints, and optimization variables

1: Initialize the algorithm parameters;

2: Generate the initial sample points within the bounds by Eq (14);

3: Evaluate the objective function for the initial sample points;

4: Construct the initial surrogate by Eqs (15)–(19);

5: Set *k*: = 1;

6: **WHILE** *k*≤*k*_max_

7:       Implement the local search near the incumbent point by Eqs (20)–(22);

8:       Evaluate the merit function of candidate solutions by Eqs (23)–(25);

9:       If $l_{max}^{in}<l_{reset}$, then reset the surrogate and go to *Step 2*; otherwise, go to the next step;

10:       Select the best candidate solution and evaluate its objective function;

11:       Update the incumbent point if the best candidate solution is better;

12:       Update the search scale according to the current searching results;

13:   Update the surrogate with the added sample point by Eqs (15)–(19);

14:       Set *k*: = *k*+1;

15: **END WHILE**

**Output:** The optimal solution.

### 3.2 Design of hierarchical power control

#### 3.2.1 Power control among different groups

Firstly, the AGC signal of the whole WF will be distributed to different groups in proportion of their regulation capacities. The regulation capacity of each group can be updated as the wind speed or direction varies, which can be determined via a maximum power optimization, as follows:

$$\max\;P_{m}=\sum_{i=1}^{n_{m}}P_{m}^{i}$$ (26)

s.t.Eqs(4)−(7)andEqs(10)−(13) (27)

where *n*_*m*_ is the number of WTs in the *m*th group; *P*_*m*_ is the total mechanical power of the *m*th group; and $P_{m}^{i}$ is the mechanical power of the *i*th WT in the *m*th group.

The maximum power optimization shown in Eqs (26) and (27) for each group is solved by DDSO, which can be executed offline with the updated wind speed and direction. Consequently, the AGC signal of each group can be written as

$$P_{m}(k)=\left\{ \begin{array}{c} P_{m}(k-1)+\Delta P(k)\times \frac{P_{m}^{up}}{\sum_{i=1}^{M}P_{i}^{up}},\;\mathrm{if}\;\Delta P(k)\geq 0\\ P_{m}(k-1)+\Delta P(k)\times \frac{P_{m}^{down}}{\sum_{i=1}^{M}P_{i}^{down}},\mathrm{otherwie} \end{array}\right.$$ (28)

$$\Delta P(k)=P_{WF}^{ref}(k)-\sum_{m=1}^{M}P_{m}(k-1)$$ (29)

$$P_{m}^{up}=P_{m}^{max}-P_{m}(k-1),m=1,2,\cdots ,M$$ (30)

$$P_{m}^{down}=-P_{m}(k-1),m=1,2,\cdots ,M$$ (31)

where $P_{m}^{max}$ is the current maximum power of the *m*th group; Δ*P*(*k*) denotes the regulation power command of the whole WF at the *k*th control interval; $P_{m}^{up}$ and $P_{m}^{down}$ denote the current regulation-up and regulation-down capacities of the *m*th group, respectively; and *M* is the number of WT groups.

#### 3.2.2 Power control among different WTs in each group

To match the objective function of the whole WF, the objective function of each group can be described as follows:

$$min\;f_{m}=\mu_{1}\Delta P_{m}^{D}+\mu_{2}\Delta \beta_{m}^{D}$$ (32)

$$\left\{ \begin{array}{c} \Delta P_{m}^{D}=|P_{m}(k)-\sum_{i=1}^{n_{m}}P_{m}^{i}(k)|\\ \Delta \beta_{m}^{D}=\sum_{i=1}^{n_{m}}{\left[\beta_{m}^{i}(k)-\beta_{m}^{i}(k-1)\right]}^{2} \end{array}\right.$$ (33)

where *f*_*m*_ is the objective function of the *m*th group; $\Delta P_{m}^{D}$ denotes the power variation of the *m*th group; $\Delta \beta_{m}^{D}$ denotes the pitch angle variation of the *m*th group; and $\beta_{m}^{i}$ is the pitch angle of the *i*th WT in the *m*th group.

The optimization in each group is also solved by DDSO, but it is executed in real-time under the dynamic AGC signals.

#### 3.2.3 Execution procedure

**Fig 5:**
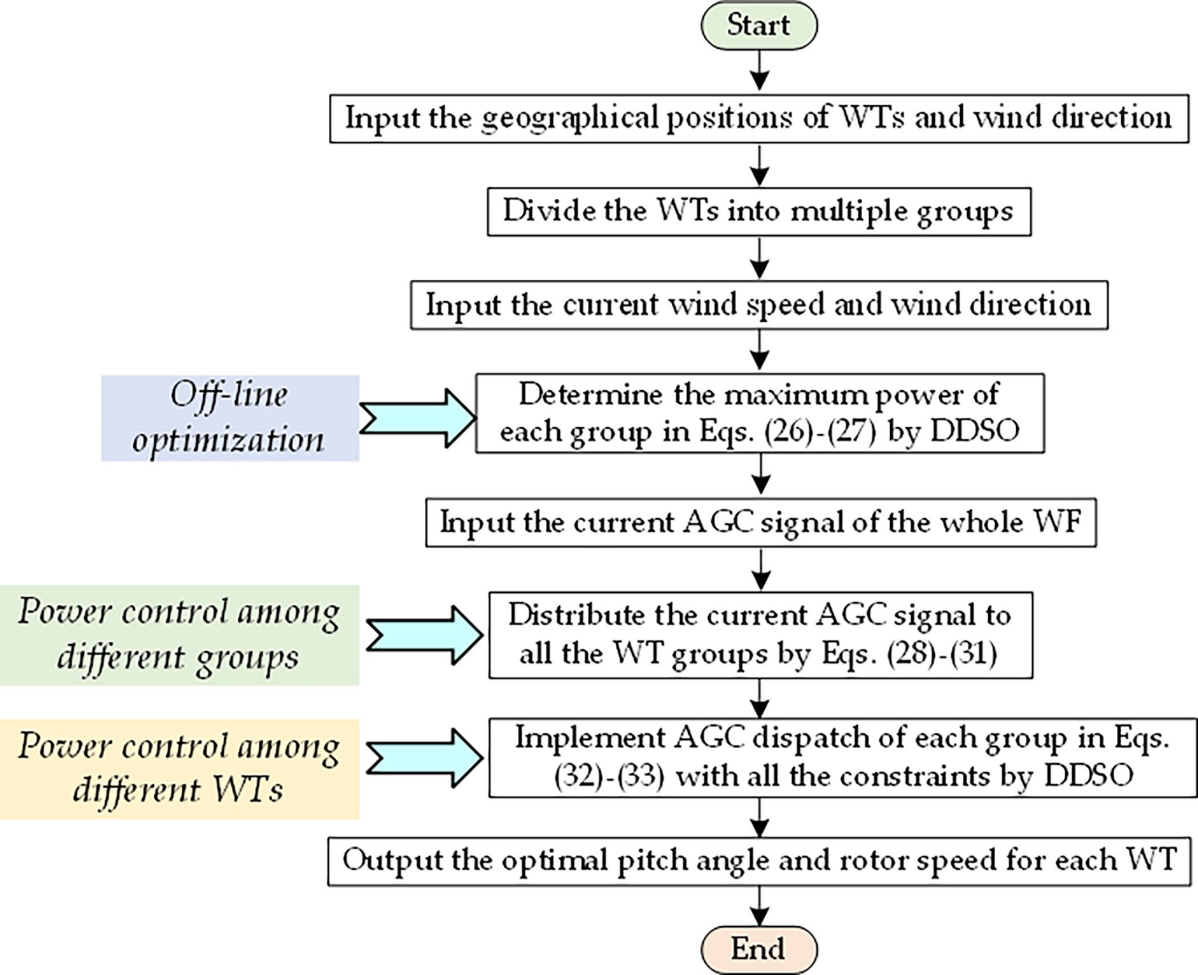
The flowchart of DDSO based HPC for a large-scale wind farm.

Taken together, the execution procedure of DDSO based HPC for a large-scale wind farm is provided in Fig 5, where the detailed execution procedure of DDSO can be found in Algorithm 1. The main novelty of this work is included in the off-line optimization for regulation capacity, the power control among different groups, and the power control among different WTs, as highlighted in Fig 5.

## 4. Case studies

**Fig 6:**
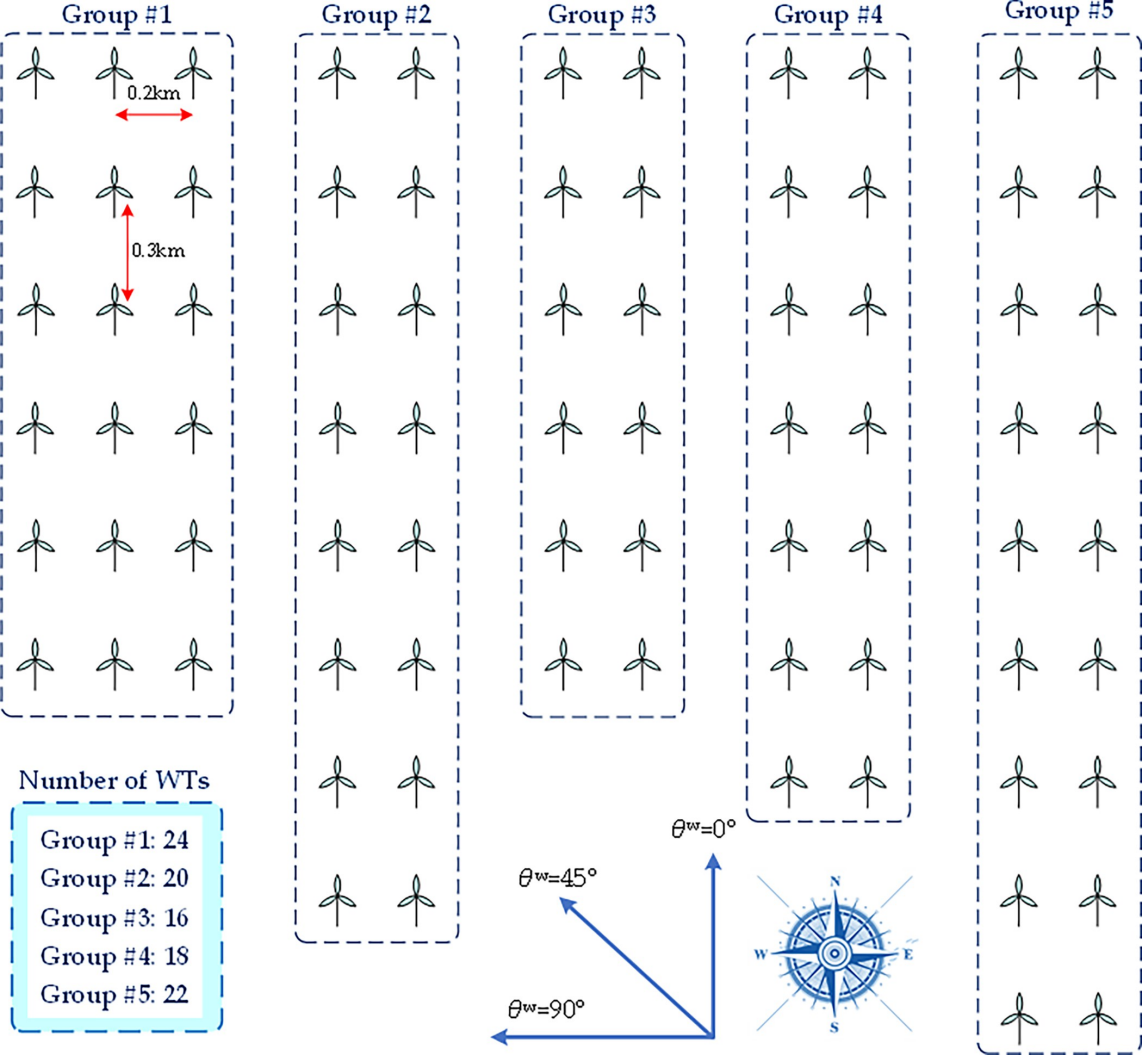
The geographical distribution of all the WTs in the designed large-scale WF.

In the case studies, a large-scale WF with 100 WTs is designed to evaluate the performance of the proposed technique, where the geographical distribution of all the WTs is given in Fig 6. Besides, the parameters of each WT are provided in Table 1. The initial pitch angle and rotor speed for each WT are set to be 0° and 3.2 rad/s, respectively. The weight coefficients *μ*_1_ and *μ*_2_ in Eq (8) are set to be 1000 and 0.001 as the power deviation is more important than pitch angle variation for AGC signal response. In order to further verify the proposed DDSO, two meta-heuristic algorithms including GA [32] and PSO [33] are adopted for performance comparison, where the main parameters of these algorithms are given in Table 2.

**Table 1:** Detailed parameters of WT.

Parameter	Symbol	Value
Rated power	*P*_rate_	1.5 MW
Air density	*ρ*	1.225 kg/m^3^
Rotor radius	*R*	30.5 m
Maximum pitch angle	*β*_max_	30°
Minimum pitch angle	*β*_min_	0°
Maximum ramp rate of the pitch angle	${\left(\frac{d\beta }{dt}\right)}_{max}$	8°/s
Minimum ramp rate of the pitch angle	${\left(\frac{d\beta }{dt}\right)}_{min}$	-8°/s
Maximum rotor speed	*ω*_max_	4 rad/s
Minimum rotor speed	*ω*_min_	2.24 rad/s

**Table 2:** Main parameters of optimization algorithms.

Algorithms	Parameters	Value
GA and PSO	Population size	50
	Maximum number of iterations	50
DDSO	Number of initial random sample points	*n*_0_ = 2*d*
	Maximum number of objective function evaluations	Online: 250 Offline: 500

### 4.1 Study of constant wind speed

**Fig 7:**
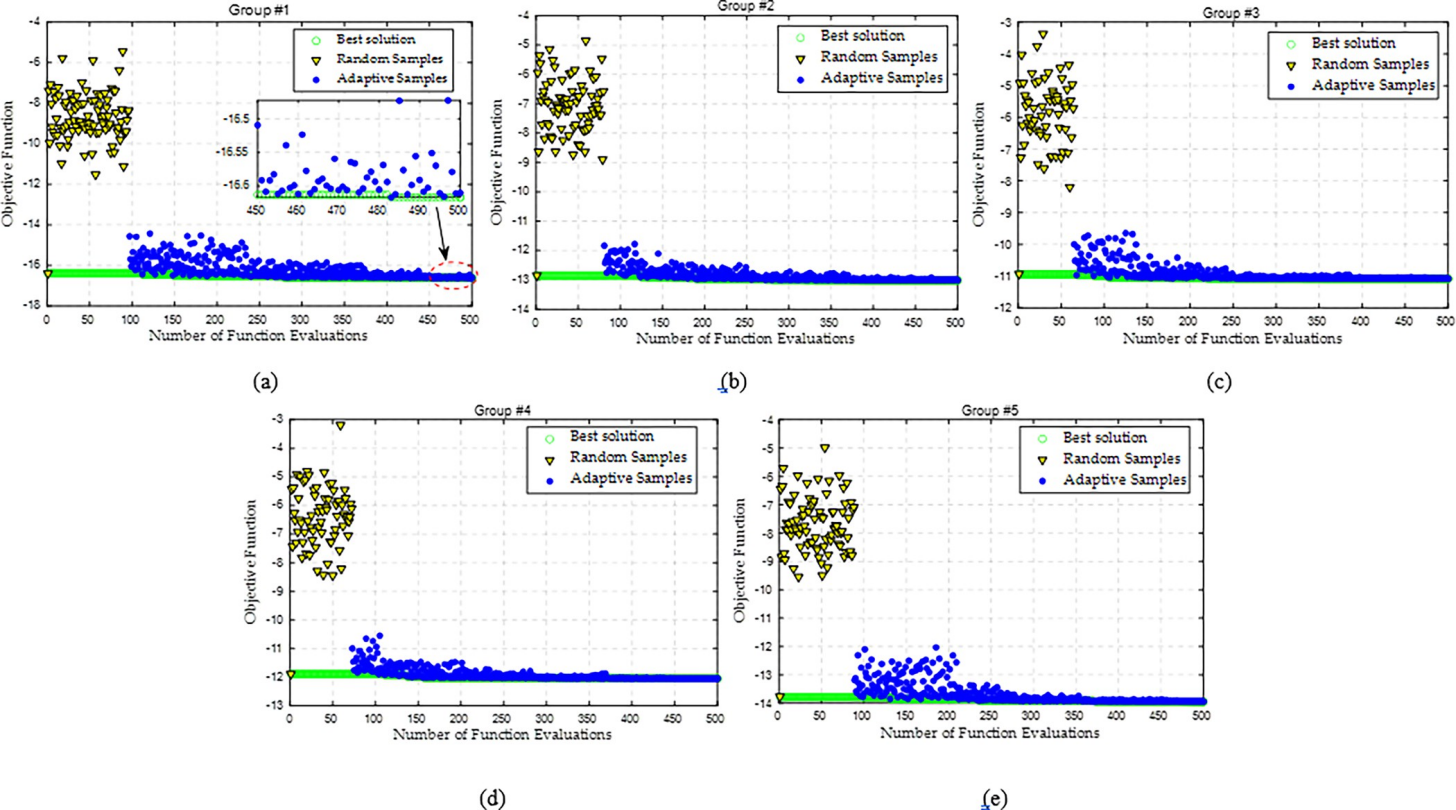
Optimization process of maximum power for each WT group by DDSO with a constant wind speed. (a) Group #1, (b) Group #2, (c) Group #3, (d) Group #4, and (e) Group #5.

In this case study, the input wind speed is set to be 12 m/s with the direction *θ* = 0°. Moreover, AGC signal for the whole WF is divided into a step signal and multiple continuous step signals. The optimization process of searching the maximum power of each WT group is given in Fig 7. It is obvious that DDSO can implement an efficient local search after the first surrogate construction based on the random samples. The obtained maximum power capacities of Groups #1 to #5 are 16.61, 13.01, 11.09, 12.05, and 13.94 MW, respectively.

#### 4.1.1 Performance under a step AGC signal

**Fig 8:**
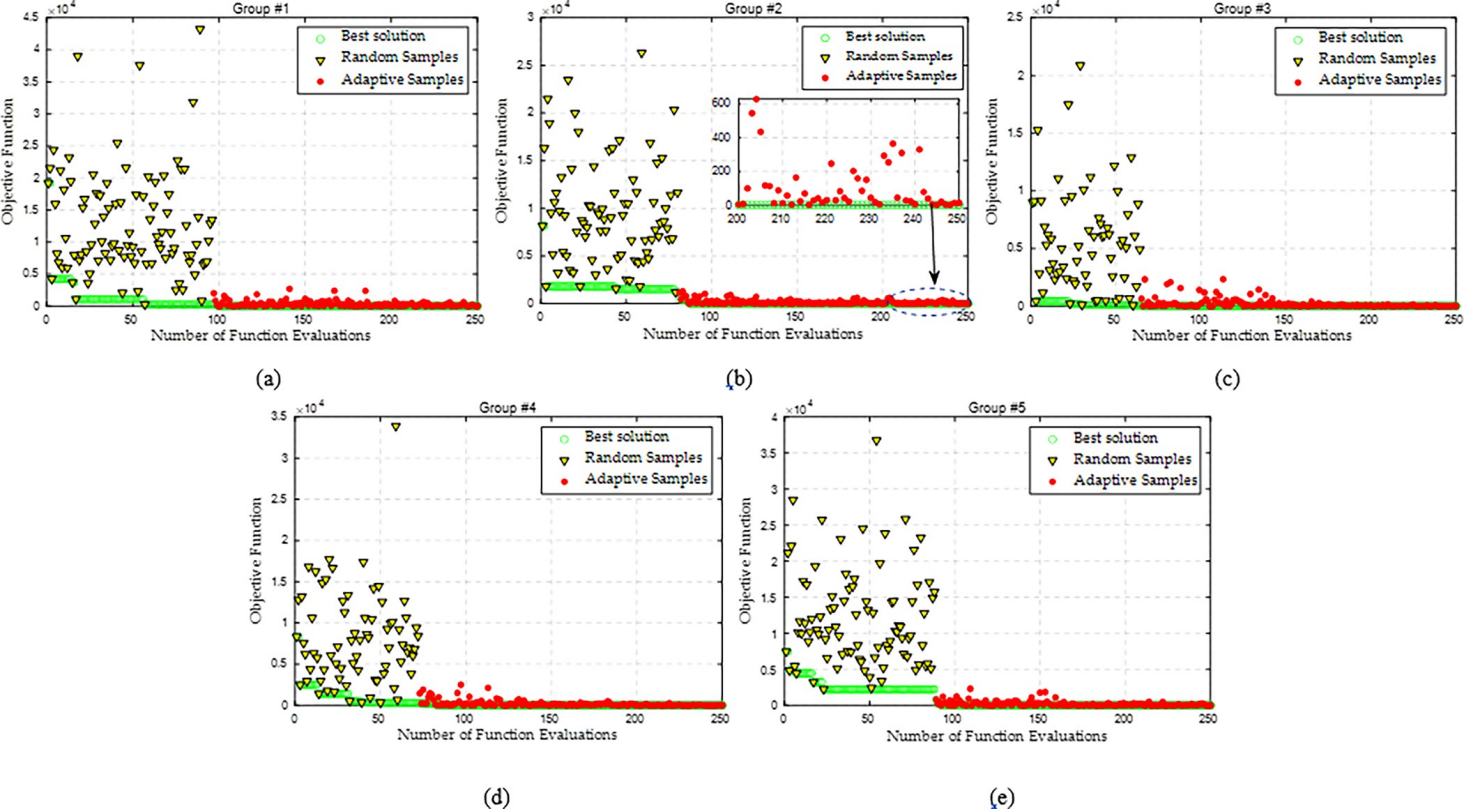
Optimization process of each WT group by DDSO for a step AGC signal with a constant wind speed. (a) Group #1, (b) Group #2, (c) Group #3, (d) Group #4, and (e) Group #5.

**Fig 9:**
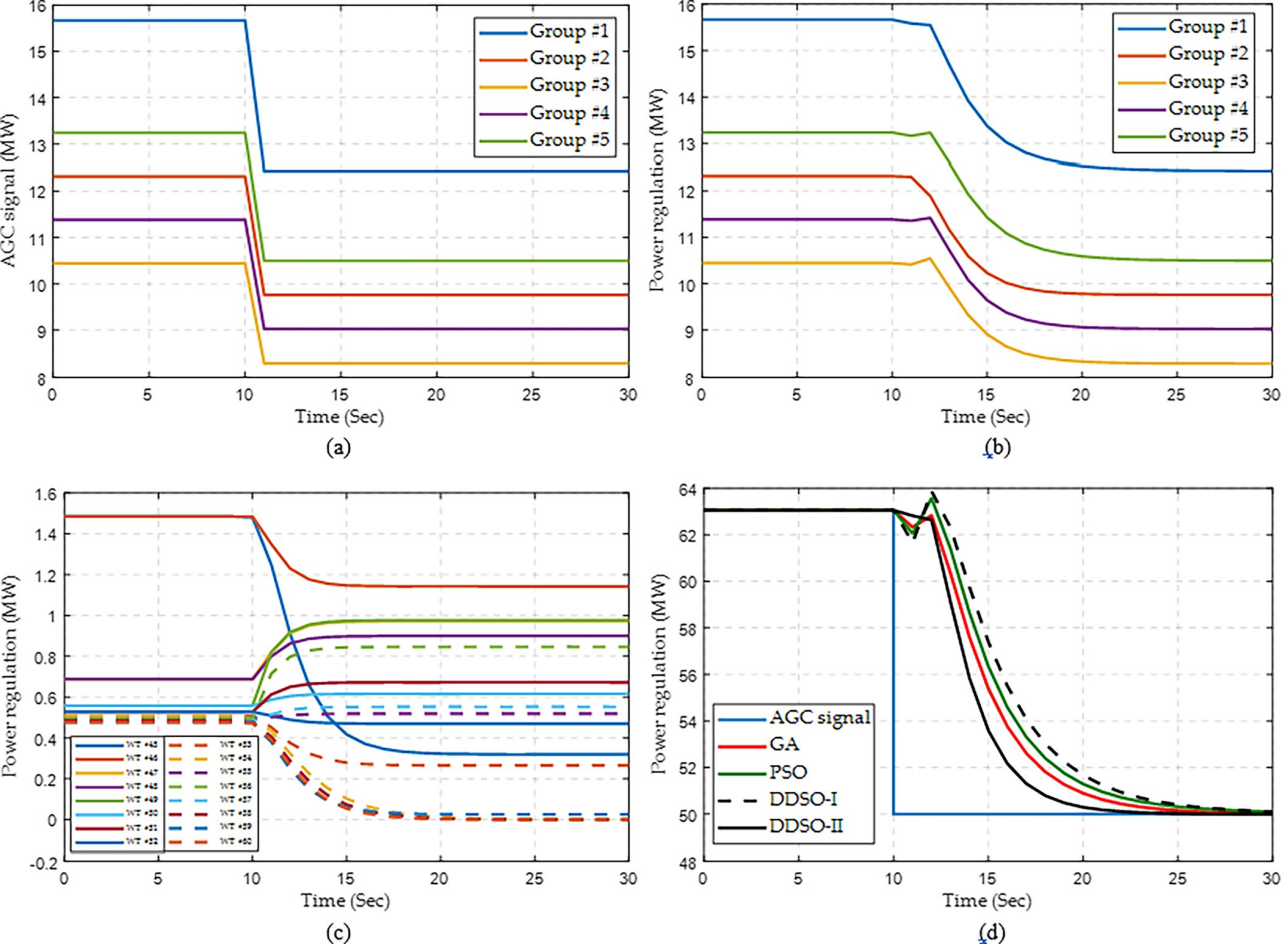
Response process of wind farm for a step AGC signal with a constant wind speed. (a) AGC signal of each group, (b) Power regulation of each group obtained by DDSO, (c) Power regulation of each WT in Group #3 obtained by DDSO, and (d) Power regulation of the whole WF.

Here, a step AGC signal for the whole WF is set to be 50 MW at the 10^th^ second. Similarly, DDSO can rapidly find a high-quality solution for each group after the random sample generation, as illustrated in Figs 8 and 9 provides the response process of wind farm for a step AGC signal with a constant wind speed, where DDSO-I denotes the DDSO without considering the pitch angle variation in Eq (8); and DDSO-II denotes the DDSO with considering the pitch angle variation. It can be found that a group with a larger regulation capacity will be assigned with a larger AGC signal, as shown in Fig 9(A). With the assigned AGC signal, each group can rapidly vary the current operation point to the expected signal, as illustrated in Fig 9(B). Meanwhile, each WT can rapidly respond to trace the assigned AGC signal, as given in Fig 9(C). Consequently, the whole WF can trace the step AGC signal accurately and rapidly, as shown in Fig 9(D). Particularly, the control performance obtained by DDSO-II is better than that obtained by other algorithms, in which the performance of DDSO-I is the worst due to the large the pitch angle variation.

#### 4.1.2 Performance under multiple step AGC signals

**Fig 10:**
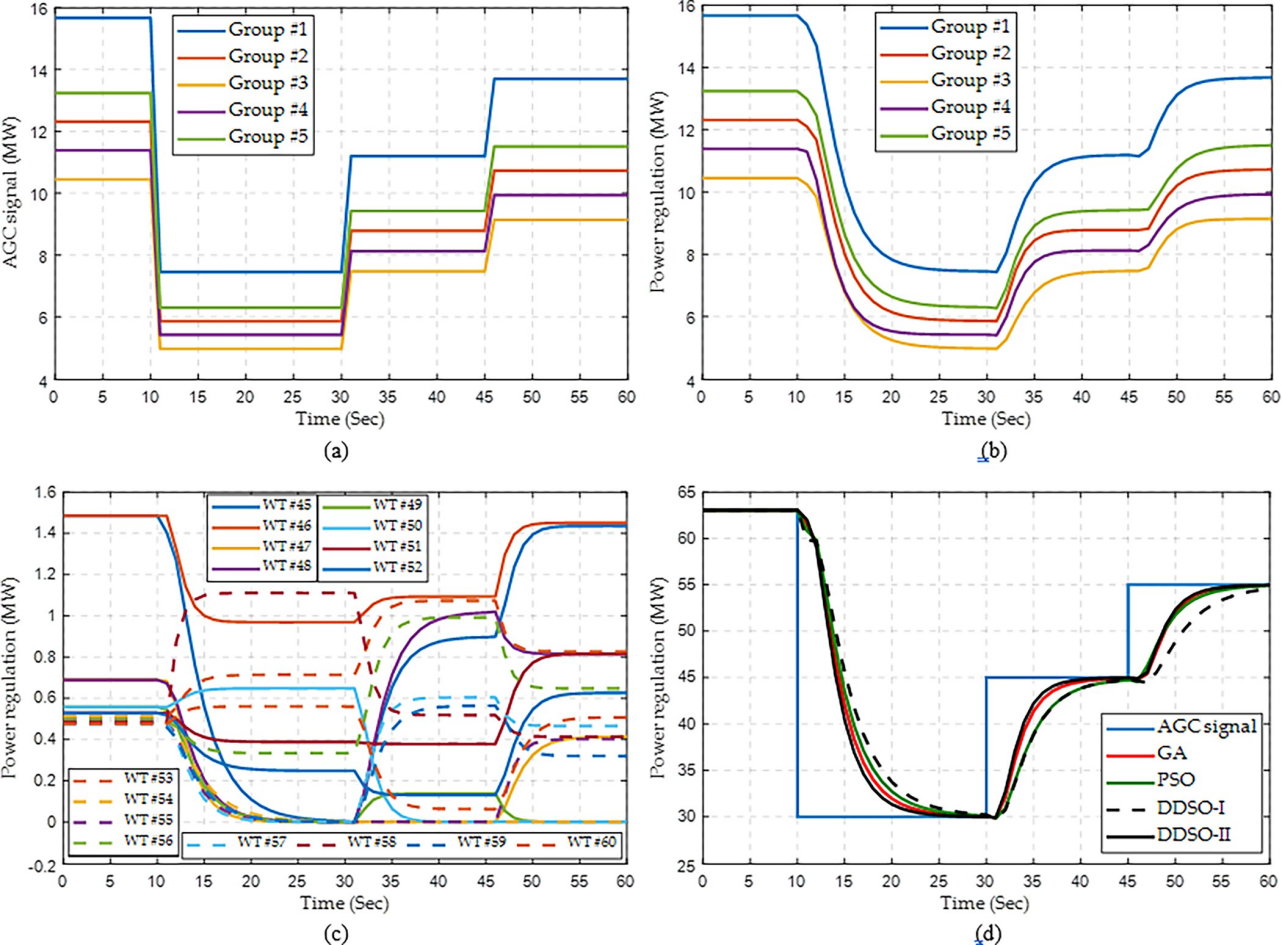
Response process of wind farm for multiple step AGC signals with a constant wind speed. (a) AGC signal of each group, (b) Power regulation of each group obtained by DDSO, (c) Power regulation of each WT in Group #3 obtained by DDSO, and (d) Power regulaiton of the whole WF.

Here, three continuous step AGC signals for the whole WF are set to be 30, 45, and 55 MW at the 10^th^, 30^th^, and 45^th^ seconds, respectively. Similarly, the assigned AGC signals of Group #1 are higher than other groups since its initial operation point and maximum power capacity are the largest, as shown in Fig 10(A). Besides, each group can rapidly trace the assigned AGC signal in Fig 10(B), which verifies the effectiveness of the power control strategy among different groups. Through implementing the optimal pitch angle and rotor speed, all the WTs can rapidly vary the current operation point to the expected AGC signal, such as the WTs of Group #3 in Fig 10(C). Finally, the power output of the whole WF can rapidly match the multiple step AGC signals, in which the response performance by DDSO-II is the best among all the algorithms, as given in Fig 10(D).

### 4.2 Study of dynamic wind speed

In this case study, the AGC signals are set to be the same as that in the case study of constant wind speed. In the meantime, the wind speed and the wind direction will vary during the power regulation for AGC.

#### 4.2.1 Performance under a step AGC signal

**Fig 11:**
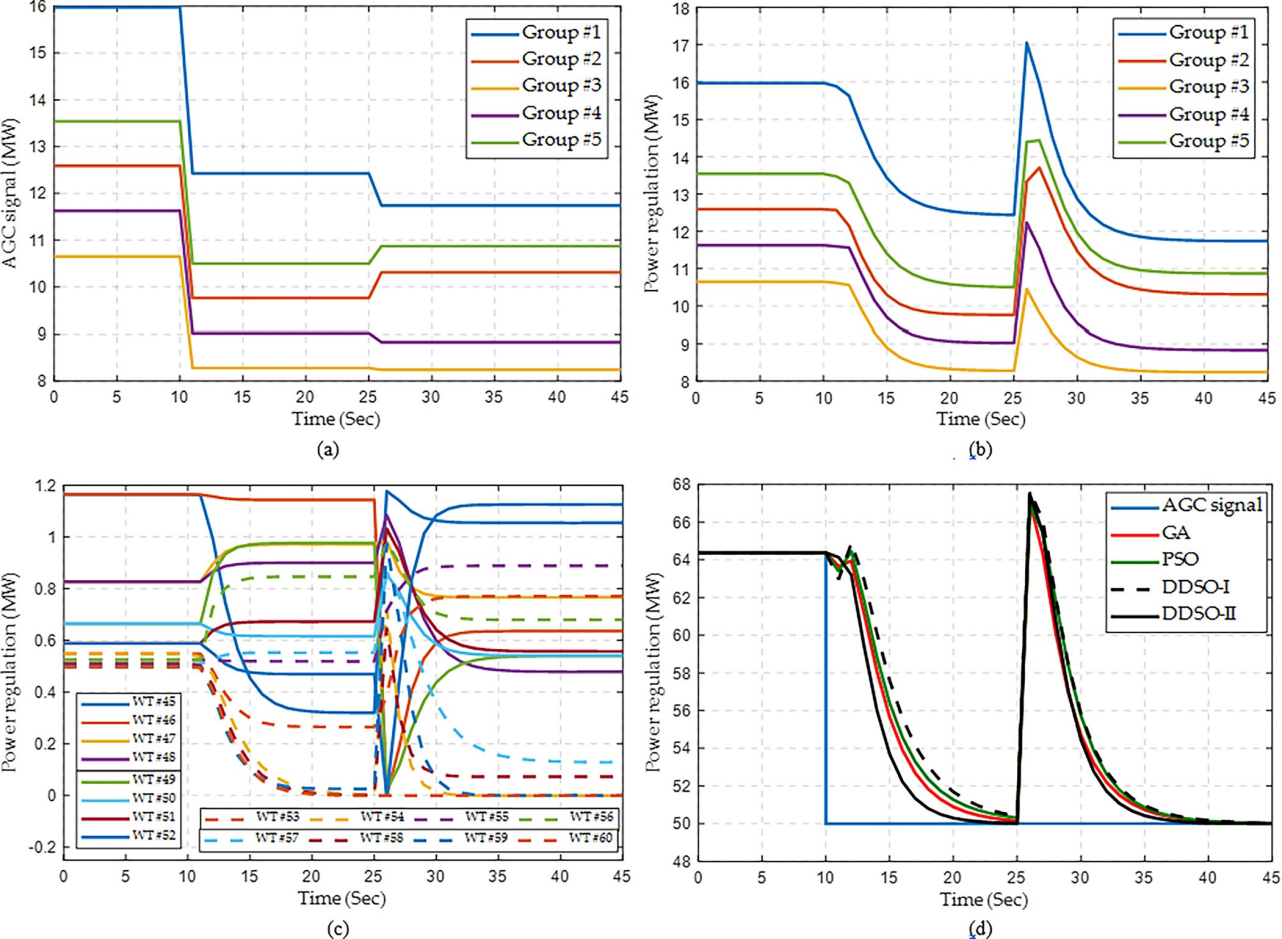
Response process of wind farm for a step AGC signal with dynamic wind speeds. (a) AGC signal of each group, (b) Power regulation of each group obtained by DDSO, (c) Power regulation of each WT in Group #3 obtained by DDSO, and (d) Power regulaiton of the whole WF.

Here, the wind speed is set to be 12 m/s, while the wind direction changes from 0° to 10° at the 25^th^ second. Although the AGC signal of the whole WF is constant after the 10^th^ second, the AGC signals to all the groups can adapt to the variation of wind direction at the 25^th^ second, as shown in Fig 11(A). When the wind direction varies, it easily leads to a large power fluctuation of each group as the wake effect between different WTs changes dramatically, as well as for the whole WF, as given in Fig 11. Nevertheless, the whole WF can be rapidly reverted to a new operation point to balance the assigned AGC signal, as well as for all the WTs (See Fig 11(C)). Finally, the response performance of the whole WF by DDSO-II is the best among all the algorithms.

#### 4.2.2 Performance under multiple step AGC signals

**Fig 12:**
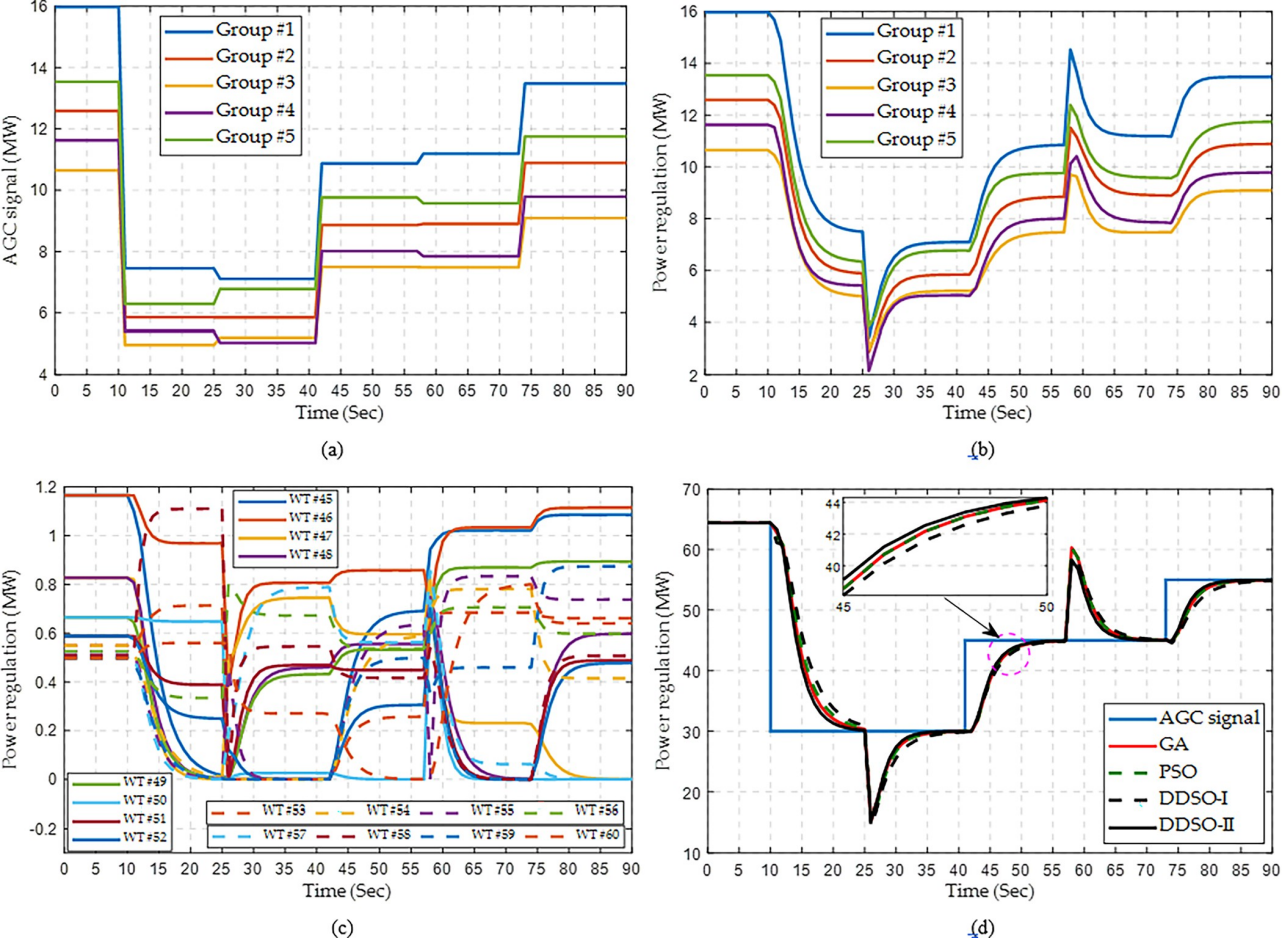
Response process of wind farm for multiple step AGC signals with dynamic wind speeds. (a) AGC signal of each group, (b) Power regulation of each group obtained by DDSO, (c) Power regulation of each WT in Group #3 obtained by DDSO, and (d) Power regulation of the whole WF.

Here, the wind speed changes from 12 m/s to 10 m/s at the 25^th^ second, and changes from 10m/s to 11 m/s at the 56^th^ second. Meanwhile, the wind direction changes from 0° to 5° at the 25^th^ second. As shown in Fig 12, the power regulation output of the whole WF can trace the assigned AGC signals well by each algorithm, while DDSO-II performs better than other algorithms. Moreover, the assigned AGC signals and power regulation outputs of all the groups can also adapt to the dynamic wind speeds, which reveals the effectiveness of the power control strategies of HPC.

### 4.3 Study of actual power deviation

Table 3 provides the actual power deviation obtained under different wind speeds with different AGC signals, where the actual power deviation can be calculated as $\sum_{k=1}^{N}|P_{WF}^{ref}(k)-P_{WF}^{act}(k)|;\;P_{WF}^{act}(k)$ is the actual power regulation output of the whole WF at the *k*th control interval; and *N* is the number of the control intervals for each case studies. Firstly, it can be found that the actual power deviation obtained by DDSO-I is the largest at each case study, which verifies that the lack of pitch angle variation will result in a large actual power deviation. Secondly, DDSO-II can acquire the smallest actual power deviation, which is only 70% of that obtained by DDSO-II under the constant wind speed with a step AGC signal. It also demonstrates that DDSO-II can acquire the higher quality dispatch schemes than that of GA and PSO within a limited number of objective function evaluations.

**Table 3:** Actual power deviation obtained under different wind speeds with different AGC signals.

Wind speed	AGC signal	GA	PSO	DDSO-I	DDSO-II
Constant	Single step	73.81	82.12	89.27	**62.49**
	Multiple step	291.86	321.25	358.91	**272.14**
Dynamic	Single step	142.68	156.09	166.93	**129.00**
	Multiple step	402.91	414.47	454.05	**367.71**

## 5. Conclusions

In summary, the main contributions of this work can be written as follows:

1. The proposed HPC can effectively reduce the optimization complexity and difficulty of power dispatch for a large-scale WF. The consideration of pitch angle variation in the objective function of HPC can make the whole WF rapidly trace the assigned AGC signal.
2. Compared with the meta-heuristic algorithms (e.g., GA), the presented DDSO can acquire a higher quality dispatch scheme for HPC within a limited number of objective function evaluations. Besides, the minimum search can guarantee an efficient local search for DDSO.
3. Four case studies under different wind speeds with different AGC signals are undertaken to verify the proposed technique. The simulation results show that the proposed technique not only can achieve an accurate and rapid trace for different AGC signals of a large-scale WF, but also can adapt to the wind speed variation by fleetly regulating the dispatch schemes. For all the case studies, the proposed DDSO-II by taking the pitch angle variation into account can acquire the smaller actual power deviations than that of GA, PSO, and DDSO-I for the whole WF. Under the constant wind speed with a step AGC signal, the actual power deviation decrement is up to 30% by DDSO-II against to DDSO-I.
